# Toads as a Tonic

**Published:** 1885-09

**Authors:** 


### Toads as a Tonic.

A Hamilton, Cal., paper says a Chinaman has devoted the whole summer and fall to gathering horned toads, which are very numerous on the red hills, and are as much dreaded as rattlesnakes. Recently he made a shipment of 2,000 of the toads to San Francisco, from which place they will be sent to China. The toads are there converted into various kinds of medicines, which sell very high. For the cure of chills and fever they are said to be the finest thing known. A toad is placed in a flask of whisky for several weeks, and then the stuff is sold as a tonic.

